# Global South-led responsible AI solutions to strengthen health systems: an emergent research landscape

**DOI:** 10.1093/oodh/oqaf016

**Published:** 2025-07-08

**Authors:** Chaitali Sinha

**Affiliations:** International Development Research Centre, 45 O'Connor Street, Ottawa, ON, Canada

**Keywords:** artificial intelligence, health equity, implementation research, Global South, gender equality, inclusion

## Abstract

Artificial intelligence (AI) solutions are being adopted across the globe, including the Global South, to address health needs and strengthen health systems. The rapid adoption of AI solutions provides tremendous potential to redress health inequities and strengthen health systems. It also entails substantial risks of deepening inequities, creating new forms of exclusion and weakening fragile health systems. Drawing on field-based case studies and interdisciplinary consultations, this paper presents an emergent research landscape that prioritizes health equity, gender equality, ethical safeguards, inclusive governance and Global South leadership. Three entry points for implementation research are proposed, which are situated within five cross-cutting prerequisites.

## INTRODUCTION

Today’s health systems are under strain from climate change, conflict, displacement, and a combination of infectious and chronic diseases. Although this strain is experienced everywhere, communities experiencing vulnerabilities across the Global South continue to face heightened, more frequent and sustained repercussions on their health and well-being [1, 2]. The COVID-19 pandemic demonstrated deep interdependencies and interconnected risks between health systems and various economic and social systems, requiring reliable solutions across sectors and jurisdictions [3]. Increases in large datasets and compute power lend themselves to artificial intelligence (AI) solutions being trained and subsequently applied to different parts of the health system [4]. These applications include health promotion, prevention, surveillance, preparedness, screening, diagnostics, treatment and control.

Alongside the promise of AI-enabled solutions in health systems, the importance of risk identification, response and human oversight required to ensure AI-enabled solutions are rights-respecting and implemented ethically, sustainably and with sound governance practices should not be ignored [5, 6]. However, not all AI models are created alike, and as with other technologies, these models reflect the knowledge, attitudes, assumptions, beliefs and biases of the designers, implementers and broader societal forces.

The role of AI in health systems has been a topic of research for many years; however, much of the research is conducted by researchers who live in the Global North [7]. Engaging researchers, decision-makers and communities across the Global South to discuss, define and develop research agendas that are relevant to their contexts can help address this imbalance [8, 9]. Moreover, the need for more implementation research to bridge the gap between the promise of AI-enabled solutions for global health and the real-world effects on health systems is gaining traction [10, 11].

## RESPONSIBLE AI FOR BETTER HEALTH

The term ‘responsible AI’ can be used to describe AI solutions that are trustworthy, ethical, human-centered, explainable, rights-respecting, privacy-preserving and secure [12]. When AI solutions are not developed or deployed responsibly, they pose significant risks to health and other legal, social, economic and cultural aspects of lives and livelihoods [13]. Designed and used responsibly, the ability of AI algorithms to analyze vast amounts of data across different sources, sectors and systems can help address root drivers of poor health, which are often based on social and digital determinants of health. However, like any solution, AI solutions are situated in complex real-world settings, which continue to operate within deeply entrenched, historically rooted and contextually specific global health challenges.

Social Determinants of Health continue to shape how individuals and groups experience health and navigate exposure to illnesses [14]. Moreover, a focus of Digital Determinants of Health, alongside the Social Determinants of Health, acknowledges that access to and use of digital tools and technologies can shape how individuals and groups experience health and access to affordable and quality health services [15]. If these contextual and co-interdependent realities are not considered in a meaningful and rigorous way, the most well-intentioned AI solution can exacerbate existing digital, health and other inequalities [16, 17].

## WHY IS IMPLEMENTATION RESEARCH NEEDED?

The role of implementation research is to explore if, how, for whom, and in what contexts interventions—from policies to programs or processes—‘work’ in the real world—and to test approaches to improve them [18]. Implementation research can help bridge the gap between the intended outcomes of promising AI solutions and their real-world adoption and performance. The need for implementation research is arguably heightened when focusing on the health needs of populations experiencing the deepest forms of vulnerabilities and deprivation. As we witness increasing numbers of AI solutions developed and deployed across the Global South, the training of these AI solutions continues to struggle with health data poverty, which is the scarcity of data that are adequately representative [19].

A growing body of research efforts led by Global South scholars are generating evidence on how responsible AI solutions can address some of these pressing—yet underrepresented—health conditions. For example, case studies featuring implementation research projects that use responsible AI solutions include tailored apps to diagnose perinatal depression in Bangladesh, improve access to ultrasound services for Indigenous women in Guatemala, and strengthen community-based surveillance of polio in Ethiopia [20]. Each case study demonstrates the local context, health challenges, responsible AI solutions and measurable results.

## EMERGENT RESEARCH LANDSCAPE

Developed based on a literature review, analysis of active implementation research projects and key informant interviews, an emergent research landscape was developed to serve as a starting point for conversations around what rigorous, locally grounded, Global-South led and inclusive implementation research should entail in the context of leveraging responsible AI to strengthen health systems. An intentional decision was made to focus on populations facing the most heightened and complex intersection of vulnerabilities and on health conditions that attract meagre investment and interest.

The emergent research landscape represents an effort to recalibrate attention away from privileged groups toward an approach that seeks to address health conditions affecting large portions of the populations who have relatively less power and influence over the global health research agenda. Examples of these population groups include women, youth, displaced people, Indigenous groups, ethnic minorities, persons living with disabilities, slum dwellers and sexual and gender minorities. Many of these groups overlap. Health conditions that they experience but receive scarce investment and attention include tuberculosis, Chagas, polio, dengue fever and malaria.

Although the landscape presented is forward-looking, it is based on examples from ongoing implementation research projects across Africa, Asia, Latin America and the Caribbean and the Middle East. There are 12 case studies presented on issues ranging from air-quality monitoring in South Africa to tackling waterborne pathogen (re) emergence in Tunisia, strengthening sexual and reproductive health knowledge among refugee women in Turkey, and promoting sexual and reproductive health for adolescents with disabilities in Ghana [20].

## INDICATIVE RESEARCH ENTRY POINTS

**Figure 1:**
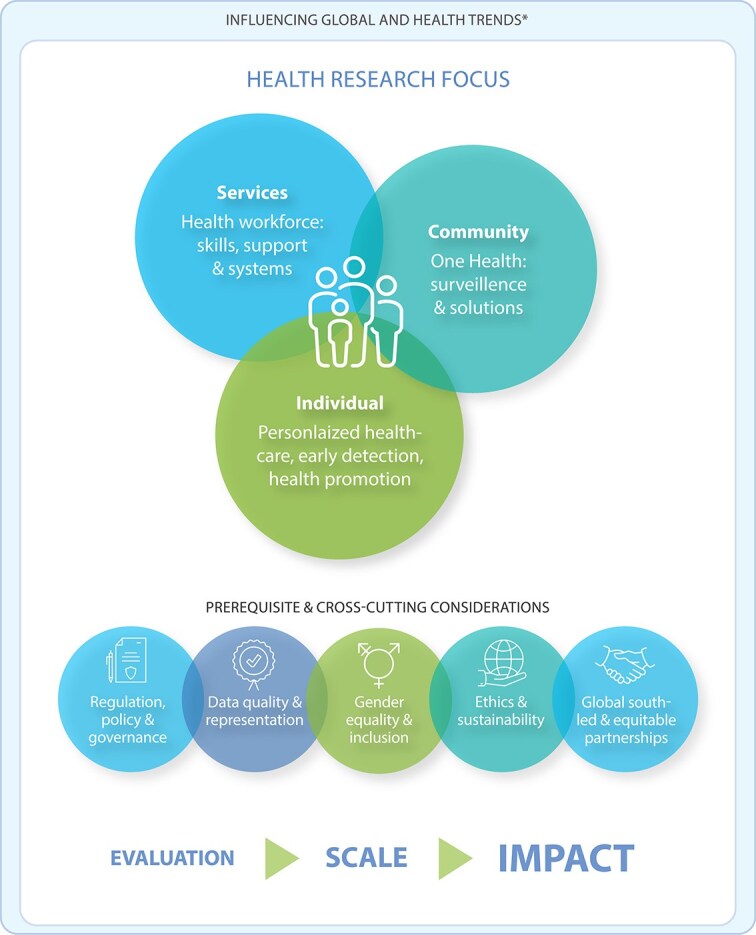
Emergent research landscape for responsible AI and global health. ^*^Global: polycrisis, growth in computational power, big data, use of AI (specifically ML), emergence of generative AI, mis- and disinformation, push back on social and gender rights, concentration of power and wealth, gender-based violence, emerging and exisiting conflicts, mass displacement, climate crisis. ***Health:*** strained health systems, digital health (including AI), self-care, preventative care, mental health, adolescent health, sexual and reproductive rights, AMR, vaccination acceptance and coverage, global health security, chronic disease, malnutrition and obesity, ERID, zoonoses, WASH, air pollution.

Three indicative research entry points emerged from the literature review, project scan and key informant interviews. Situated under three broad categories—health services, community health and individual health—the three entry points under each category are: (i) strengthening the health workforce; (ii) community-based One Health surveillance and solutions and (iii) self-care interventions, respectively. As shown in Fig. 1, there are connections and overlaps across each of these categories and their respective entry points.

### Health services: Strengthening the health workforce

The health workforce represents a diverse group of individuals who are often the first point of contact for patients, whether at a hospital, community clinic or home visit. Regardless of where these individuals are working, they can be thought of as ‘the human face’ of health systems. Whether interactions with the health workforce are in-person or via digital device, the positive, negative and mixed implications of using AI-enabled solutions for health workers is an issue that merits considerable attention and contextualized implementation strategies [21].

Many health systems in the Global South face severe shortages of qualified frontline workers [22]. Predominantly female, these workers often face barriers to training, leadership and fair pay [22, 23]. Building evidence on gender-responsive and responsible AI to support these workers is crucial for current demands, surge capacity and future preparedness. Some areas of inquiry that could be examined via implementation research include:

- Reducing the gaps in required skills and training through customized curricula.
- Exploring ways to strengthen workforce diversity to address the population’s needs with tools and through people who can deliver respectful and quality care.
- Developing and testing tools that strengthen positive and empathetic relationships between providers and patients.
- Developing AI-enabled solutions for workforce planning, especially in the context of addressing surge capacity.

### Community: One health surveillance and solutions

The COVID-19 pandemic underscored the need for stronger disease surveillance, the rising influence of zoonosis and the need for more investment in One Health approaches. As interactions between humans, animals and the environment grow in complexity, AI solutions are increasingly being considered and used to address these One Health challenges using large data-driven algorithms, data modeling and a variety of sensors [24, 25].

Effective public health surveillance, conducted across global, regional and national scales fundamentally relies on robust and timely community-based processes. A community based One Health surveillance model fosters active community participation in safeguarding both human and environmental health [26]. Notably, these health systems are iteratively strengthened through multi-level feedback loops, enhancing the design and implementation of social, technical, political and other relevant processes at the community level. Some areas of inquiry that could be examined via implementation research include:

- Identifying and analyzing models that effectively leverage institutional and citizen sources.
- Investigating interdisciplinary integration, from framework development to communications, collaboration and data sharing.
- Strengthening early warning and detection systems.
- Using sensors and other types of cost-effective, real-time monitoring of animals, humans, water sources and insects.

### Individual: Self-care interventions

More than 400 million people around the world lack access to essential health services [27]. The role of self-care for those without access to care represents ‘the ability of individuals, families and communities to promote health, prevent disease, maintain health and cope with illness and disability with or without the support of a health worker’ [6]. Examples of self-care include health promotion, disease prevention, treatment, rehabilitation and palliative care [28].

The combination of healthcare worker shortages and increased use of AI-enabled applications have led to a rise in tailored, personalized care [29]. Three non-exclusive categories of self-care proposed in this emergent research landscape include personalized health care, preventative health and early detection and health promotion. These maps loosely to the three categories in the WHO guideline: self-management, self-testing and self-awareness.

Well-designed, locally relevant and privacy-protecting responsible AI solutions can significantly improve health management and monitoring and empower individuals with personalized, proactive, person-centered and affordable care solutions. Some areas of inquiry that could be examined via implementation research include:

- Examining how users engage with AI-enabled self-care tools.
- Measuring the nature, extent and sustainability of changes in behaviors resulting from self-care interventions.
- Exploring the cost-effectiveness of AI-based self-care in resource-limited contexts.
- Exploring how AI-enabled self-care solutions can support decision-making processes among individuals with varying levels of health literacy.

These three categories and associated research entry points are not meant to provide a comprehensive or exhaustive case for being the most important issues to address. They are meant to show indicative levels in the health system, different issues to be addressed and examples of potential knowledge gaps to be filled with implementation research.

## CROSS-CUTTING PREREQUISITES

The five prerequisites included in the emergent research landscape are expected to be considered regardless of the health entry point, proposed AI solution, population(s) of interest or intended outcomes. Revisiting these prerequisites and making corrections to the research process is required. They include:

*Regulation, policy and governance* are critical considerations to ensure the safe, ethical and effective use of AI in health care, as they provide frameworks for accountability, privacy protection and equitable access [30]. The rapid pace of evolution and deployment of AI-enabled solutions underline the urgency to achieve a balance between fostering innovation and maintaining regulatory compliance that strengthens safety and protects rights [31]. Moreover, the cross-sector and cross-jurisdiction flow of data merit discussions about AI regulation, policy and governance at the national and international levels [32].

Some issues to consider under this pre-requisite include:

- Examining the existence of data protection laws
- Strengthening regulations to ensure AI systems are transparent and explainable
- Ensuring clear lines of responsibility and accountability for AI-enabled decisions in health systems

*Data quality and representation* is the foundation for equitable, fair and responsive health systems. Poor data quality can result from under-representation, misrepresentation and over-representation, with each resulting in different forms of biases [33]. Recognizing that the use of AI inherently entails data limitations that will lead to some level of bias [34], there are ways to detect and limit undesirable biases—from selection bias to exclusion bias, detection bias and others [35]. Large language models (LLMs) and large multimodal models (LMMs) offer personalized responses but vary in accuracy across languages and populations due to training data skews, in particular with low-resource languages [36].

Some issues to consider under this prerequisite include:

- Strengthening ontologies and data structures
- Measuring the availability and reliability of local datasets to address groups most impacted
- Exploring how cross-jurisdictional data sharing can be broached while respecting local and global data governance standards

*Gender equality and inclusion* cannot be excluded, as we have long known that groups experiencing vulnerabilities consistently face greater health risks, less healthcare access and less favorable health outcomes [37]. Recognizing health outcome disparities among women, men and gender-diverse individuals stems from biological, social, cultural and political factors. In addition, a failure to meaningfully address this reality turns a blind eye to how gender relations and different forms of exclusion influences access to resources such as health information, health services, digital health literacy, digital devices, digital infrastructure and connectivity.

The heightened use of AI solutions can perpetuate and amplify coded inequity, meaning the individuals who design and adopt AI tools do not think carefully about different forms of systemic oppression and exclusion [38]. The ability of AI-enabled systems to work with large datasets, which may or may not include disaggregated data, should not be conflated with assumptions that these systems are examining issues of exclusion or that they will use a lens of intersectionality to examine different aspects of identity and experience influencing exposure to health-related risks or access to health services [39].

Some issues to consider include:

- Considering access to devices, connectivity and other resources to leverage AI-enabled solutions
- Diversifying the pool of locally trained talent to design, deploy and use AI-enabled solutions
- Analyzing how social and gender norms influence AI regulations, datasets, algorithms, use and health-seeking behaviors

*Ethics and sustainability* in AI for healthcare is a critical area for study [40, 41] but tends to focus on Global North contexts and clinical settings [42]. The WHO outlines six core AI ethics principles: protecting autonomy, promoting wellbeing and safety, ensuring transparency, fostering responsibility, ensuring inclusiveness and promoting responsive and sustainable AI [30]. As AI uses more detailed data, protecting individual and group privacy becomes critical, given instances of data misuse [43]. Ethical dilemmas arise from both AI application and its withholding, potentially increasing harm and inequalities. Responsible AI must consider ethical principles and sustainability across social, economic, environmental, political and technological dimensions.

Some issues to consider include:

- Aligning the design, implementation and assessment of AI solutions with human rights principles, including minimizing bias to enhance fairness across different groups
- Ensuring transparency, explainability and autonomy across AI models
- Addressing the growing concerns related to the environmental impact of AI

*Global South-led leadership and equitable partnerships* are distinct concepts but interconnected in how they are conceived, upheld and operationalized. Strong, sustained research leadership from the Global South, generating locally relevant evidence for policy and practice, is critical for robust and responsive health research systems [44]. This leadership is part of a broader push for equitable partnerships, which ensures contextual understanding, local expertise and community connections are leveraged [45].

The importance of co-creation and Global South leadership could not be more fitting for the current AI and global health environment. The stakes are high and the fallout of being excluded from discussions related to governing and regulating AI would be borne disproportionately by individuals experiencing the deepest forms of inequalities across the Global South.

Some issues to consider include:

- Strengthening Global South research capacities and local talent developing AI solutions for global health
- Ensuring principles of equitable partnerships are upheld
- Supporting evidence published with lead authorship from Global South scholars

The net impact—positive, negative or mixed—of AI solutions can be challenging to manage if their repercussions are not carefully considered and meaningfully assessed with lessons drawn from them. This points to the need for evaluation frameworks and metrics to assess and learn from various stages of AI development, deployment, integration and adoption into health systems [46]. For example, establishing systematic benchmarking and standards to evaluate the safety and effectiveness of AI algorithms when used for clinical or public health [47].

## CONCLUSION

This paper highlights the promise and complexity of leveraging responsible AI to strengthen health systems in the Global South. Amid rapid AI adoption, particularly in low-resource settings, the risks of reinforcing health inequities are significant if ethical, inclusive and equity-focused safeguards are not in place. Drawing from an emergent research landscape report, this paper proposes three research entry points—health workforce strengthening, community-based One Health surveillance, and self-care interventions—grounded in five cross-cutting prerequisites. This framework highlights the need for implementation research that is rigorous, inclusive, locally grounded and led by Global South actors. As this field evolves, investments in Global South-led research and co-creation are essential to ensure that AI serves as a tool to strengthen, not strain, health systems. This implementation research landscape and proposed entry points offer a starting point for shaping a more just and responsive future for stronger health systems that leverage responsible AI-enabled solutions.

## Data Availability

There are no new data associated with this article.
